# *Clostridioides difficile* in equidae necropsied in Northwestern France, between 2019 and 2021

**DOI:** 10.1128/spectrum.02165-25

**Published:** 2025-12-30

**Authors:** Sandrine Petry, Jackie Tapprest, Karine Maillard, Frédéric Barbut, Fabien Duquesne, Sofia Kozak, Nathalie Foucher, Maud Bernez-Romand, Ludovic Bridoux, Isabelle Poquet

**Affiliations:** 1 ANSES, Normandy Laboratory for Animal Health, Physiopathology and Epidemiology of Equine Diseases Unit (PhEED) 505178 , Goustranville, Normandy, France; 2 LABÉO Frank Duncombe, Caen, France; 3 National Reference Laboratory for C. difficile, Hôpital Saint Antoine, Assistance Publique Hôpitaux de Paris (AP-HP) 26930, Paris, France; 4 INSERM S-1139, Université Paris-Cité, Paris, France; 5 Université Paris-Saclay, INRAE, AgroParisTech, Micalis Institute, Jouy-en-Josas, France; Tainan Hospital Ministry of Health and Welfare, Tainan, Taiwan

**Keywords:** *Clostridioides difficile*, One Health, necropsied Equidae, prevalence, diversity, toxin detection

## Abstract

*Clostridioides difficile,* an anaerobic, spore-forming enteropathogen, is less studied in animals than in humans despite its importance in One Health. We evaluated *C. difficile* occurrence, diversity, circulation, and virulence in French Equidae (*n* = 100) after their necropsy in northwestern France, from 2019 to 2021. We systematically recovered all cecal contents and any watery intestinal contents. We isolated *C. difficile* strains, determined their toxin gene profile by PCR, and established their PCR-ribotype according to the WEBRIBO database. We also performed free toxin detection. Twenty-seven Equidae were positive for *C. difficile* and 20 had a toxigenic strain, including one animal co-colonized by a non-toxigenic strain. Toxigenic isolates belonged to eight already described ribotypes: (i) 078 and 126 (toxin genes of wild-type size: *tcdA^+^ tcdB^+^ cdt^+^*), (ii) 005, 012, 020, AI-53, and FR227 (*tcdA^+^ tcdB^+^*), and (iii) 017 (*tcdB^+^*). Non-toxigenic isolates were of ribotypes 009, 035, and 439. Ribotypes 017 and 009 were predominant (*n* = 5). In two cases, Equidae of the same premises shared the same ribotype, either 020 or 009. Free toxins were detected in four animals: they displayed signs of diarrhea and a *C. difficile* of ribotype 126 (*n* = 1) or 017 (*n* = 3) as the only detected enteropathogen, suggesting a *C. difficile* infection (CDI). Three of them had received antibiotics. Two had died from an entero-toxic infection, for which *C. difficile* ribotype 017 was the only identified cause. French Equidae displayed common pathogenic *C. difficile,* and ribotype 017 was highly virulent. These findings are of concern from a One Health perspective.

**IMPORTANCE:**

*C. difficile*, a major enteropathogen widely disseminated in the environment, is a One Health issue. Animals are raising concern as human contamination sources. Equidae are in close contact with humans and also develop *post*-antibiotic and healthcare-associated CDIs. The systematic survey of Equidae necropsied from 2019 to 2021 in the leading horse breeding region in France revealed that 20% harbored pathogenic strains. These belonged to clinically important ribotypes, raising the possibility of cross-species, zoonotic or anthropo-zoonotic transmission. Free toxins, which are rarely tested in animals, were detected in four animals with signs of diarrhea and a toxigenic *C. difficile* as the only identified enteropathogen, suggesting CDI. In two of them, *C. difficile* ribotype 017 was the only identified cause of entero-toxic disease and death. French Equidae could play a role in the dissemination of pathogenic *C. difficile* and notably ribotype 017. They should be surveilled carefully from a One Health perspective.

## INTRODUCTION

*Clostridioides difficile* is a spore-forming anaerobe responsible for diarrhea and colitis (1, 2). *C. difficile,* whose spores are transmitted by the fecal-oral route, is a major cause of healthcare-associated diarrhea and a rising cause of community-acquired infections (1, 2). *C. difficile* infections (CDIs) can be severe, with complications and recurrences, and life threatening (1, 2). Toxin A (TcdA) and Toxin B (TcdB) are responsible for symptoms, with TcdB alone being sufficient to cause disease. The binary toxin (CDT) has an accessory role (3). Non-toxigenic strains are unable to cause disease, while toxigenic strains can cause disease or be asymptomatically carried (4). Antibiotics, hospitalization, and age increase the risk of developing CDI (1, 2). Antibiotics notably induce microbiota dysbiosis, which favors spore germination, vegetative multiplication, and ultimately, toxin production (1, 2). Healthcare-associated CDIs are treated with fidaxomicin and vancomycin (2, 5), which can contribute to microbiota dysbiosis and favor *C. difficile* re-emergence, possibly from persisting biofilms (2, 6, 7). In this context, the last resort treatment is fecal microbiota transplant (FMT) (5).

*C. difficile* is a One Health issue (8–11). Spores, which are highly resistant, are widely disseminated by fecal spreading in the environment and can contaminate different hosts (8–12). *C. difficile* strains can be transmitted between host species (11, 13–15). Toxigenic strains can cause CDI in a wide range of wild, farm, and companion animals (9–12, 16, 17). Equidae, which are monogastric mammals, are closely interacting with humans and relevant to zoonotic and anthropo-zoonotic transmission. Importantly, in these animals of high economic value (French Institute for Horse and Riding [IFCE], https://www.ifce.fr), antibiotics treatments and hospitalization are common and increase the risk of developing CDI, as it is the case in humans. In Equidae*, C. difficile* is also responsible for outbreaks and sporadic cases of CDI (18–22). Both Equidae and humans are treated with vancomycin and have long been treated with metronidazole, which is still used in animals but replaced with fidaxomicin for hospitalized patients (2, 5). Like humans, Equidae can also be treated with FMT (23). The main difference between Equidae and humans as *C. difficile-*infected populations is age. While <3-years-old children are mainly asymptomatic carriers (4), CDI is common in foals (18, 19).

Data about the importance of *C. difficile* in horses (8, 10, 12, 20, 24) remain scarce in France. Here, we studied *C. difficile* in Equidae necropsied in Normandy, the leading horse breeding region in France (12,600 foals and 145,000 Equidae in 2021 according to IFCE: https://statscartes.ifce.fr/storage/files/1/pdf/IFCE-Depliant-chiffres-cles-2022-WEB.pdf). Necropsy (25) provided the opportunity both to recover digestive contents from a population with diverse clinical pictures and to get access to detailed animal data including the cause of animal death. We included all necropsied Equidae, whatever the cause of death and clinical picture, to test the presence of *C. difficile* and that of toxins when relevant. Our objective was to *post-mortem* evaluate *C. difficile* occurrence, diversity, putative circulation, and virulence in French Equidae.

## MATERIALS AND METHODS

### Equine necropsy

At the French National Agency for Food, Environmental and Occupational Health & Safety (ANSES), the PhEED Unit in Normandy is in charge of surveillance, prevention, and study of equine diseases. The veterinarian of PhEED is notably in charge of equine necropsies at the request of breeders or veterinarians to determine the cause of animal death. The standardized procedure includes examination of the cadaver, evisceration, and organ examination. Detailed animal data including identification (necropsy date, sex, age, breed, and location), *ante-mortem* information (treatments, including antibiotics for 2–3 weeks before death, and hospitalization) and *post-mortem* data (necropsy observations, standard analyses performed in case of signs of infection to identify pathogens, and the cause of animal death) are registered (https://sitesv2.anses.fr/fr/minisite/resumeq/presentation-de-resumeq) (25) (Table S1). Housing style was not recorded.

In 2018, we chose five necropsied Equidae with *post-mortem* signs of endo-enterotoxaemia and diarrhea and collected their watery intestinal contents for a pilot study. Then, from May 2019 to August 2021, we studied all (*n* = 100) necropsied animals (except fetus and stillborn foals), irrespective of clinical picture or cause of death. This total number, after 2 years characterized by Covid-19 and lockdowns, was relatively low, matching the previous annual average. Animals originated from Normandy (*n* = 97), Brittany (*n* = 1), and Centre Val de Loire (*n* = 1) (the origin of one animal was not registered) (Table S1). Animal digestive contents were systematically recovered in the cecum (CAE; *n* = 100), and in case of a watery consistency, in any other intestine segment (CDL; *n* = 30), notably the colon. Aliquots of all digestive contents except a cecal one (129 samples in 2019–2021) were preserved at 4°C, −20°C, and −70°C.

This study followed institutional guidelines and was approved by the scientific committee of IFCE that funded the project. All data are anonymous.

### Standard pathogen testing

In case of *post-mortem* observed signs of infection, the veterinarian requested routine pathogen testing in relevant samples (Table S1). For intestinal infections, usual testing in digestive contents was as follows. *Escherichia coli* colonies, after detection on Eosin Methylene Blue plates, were streaked on blood plates to see hemolysis. *Salmonella* spp. were isolated on XLD plates (Thermo Scientific). Clostridial species were detected under anaerobiosis: *C. difficile* on ChromID (BioMérieux), *Clostridium perfringens* and *Paeniclostridium sordellii* on Columbia agar containing 5% sheep blood (BioMérieux). Matrix-Assisted Laser Desorption/Ionization Time-Of-Flight (MALDI-TOF) Mass Spectrometry (MS) (MBT Smart mass spectrometer, Compass and FlexControl softwares, Bruker Daltonics) confirmed identification. Rotavirus was identified by PCR. Intestinal parasite communities were identified and quantified by visual inspection (26).

### *C. difficile* testing, isolation, and characterization

*C. difficile* presence was independently tested by streaking a sample of any digestive content onto ChromID or CLO plates (BioMérieux) and incubating for 48 h at 37°C under anaerobiosis. In case of a negative result, a 1 g-aliquot was harvested after sample homogenization, inoculated into a rich, permissive medium: BHI broth supplemented by cysteine (0.1%), yeast extract (5 g/L), taurocholate (1 g/L), D-cycloserine (250 µg/mL), and cefoxitin (8 µg/mL) (OXOID), and finally incubated for 3–7 days (enrichment) before plating onto ChromID. A positive stool was included as a control. Suspicious, irregular, and/or black colonies were screened by PCRs targeting *tpi*, *tcdA,* and *tcdB* genes (27, 28). Identification was confirmed by MALDI-TOF MS. A single *C. difficile* colony per positive sample was then grown in BHI broth at 37°C under anaerobiosis to be preserved in glycerol (17%) at −70°C, except in two cases: two strains (non-toxigenic in animal #48 and toxigenic in #54) characterized without being preserved, could not be recovered later.

All preserved isolates (*N* = 34 from *n* = 25 Equidae in 2019–2021 and *N* = 1 in 2018) were characterized by multiplex PCR targeting: (i) *tpi* gene, (ii) wild-type or truncated *tcd* and *cdt* genes, and (iii) *lok* fragment characterizing non-toxigenic strains, followed by capillary gel electrophoresis (29). For ribotyping, PCR profiles after capillary gel-based electrophoresis (30) were analyzed with GeneMapper software (Thermo Fischer Scientific, Villebon-sur-Yvette, France) and ribotypes assigned according to the WEBRIBO database (https://webribo.ages.at/). The first isolates were characterized by Multi Locus Sequence Typing (MLST) (31).

### Susceptibility to vancomycin and to metronidazole

The Minimum Inhibitory Concentration (MIC) was determined using Etest (BioMérieux) according to the EUCAST and CA-SFM recommendations when the experiments were performed (https://www.sfm-microbiologie.org/wp-content/uploads/2021/04/CASFM2021__V1.0.AVRIL_2021.pdf). Briefly, a suspension of 1.0 MacFarland was prepared in Schaedler broth and spread on a pre-reduced Brucella blood agar (Becton Dickinson) supplemented with vitamin K1 (10 mg/L), hemin (5 mg/L), and defibrinated horse blood (5%) (OXOID). Of note, Brucella blood agar medium was recently published to be suboptimal for Metronidazole Etests (32). A strip, in which antibiotic concentration ranged from 0.0016 to 256 mg/L, was laid on the plates, which were then incubated for 24h (vancomycin) or 48h (metronidazole) at 37°C under anaerobiosis (using Thermo Scientific Oxoid AnaeroGen Compact sachets). We included *C. difficile* reference strain ATCC 700057 as a control.

### Toxin detection

*C. difficile* toxin A, toxin B, and glutamate dehydrogenase (GDH) were detected in digestive contents using an Enzymatic Immuno-Assay (Quick Check Complete, Alere) according to manufacturer’s instructions. For each newly opened kit, the supplied positive control was tested. For each series of experiments, we also included an aliquot of a human stool known to be positive and stored at −80°C. A second independent observation confirmed any weak positive result.

### Statistical analysis

We used a Fisher’s exact test to analyze the equine population composition, after excluding unknown values, and a Mann-Whitney test to compare strain MICs.

## RESULTS

### Necropsied equidae

We studied all Equidae (*n* = 100) necropsied at ANSES in Normandy from May 2019 to August 2021, without any selection (Table S1). They belonged to 78 premises, all located in Normandy except three (Table S1). Most were females (67%) and <1-year-old animals (59%), with foals (< 6 months) representing half of the population (48%) (Table 1). Nine breeds were present, mostly Thoroughbred (42%), Trotters (31%), and Saddlebred horses (11%) (Table 1). Animals had died from very diverse (> 30) causes including infectious or non-infectious diseases and trauma (Table S1). The first and second most prevalent causes of death were (i) endo-enterotoxaemia (12%), an enteric disease caused by toxin-producing bacteria like Salmonellae and Clostridia (21), and (ii) infection by *Rhodococcus equi* (9%), common in foals (33).

**TABLE 1:** Equine population: data summary^*a*^

*C. difficile*	Presence			Pathogenicity				Toxin production		
	No	Yes	*P*-value	Non-toxigenic	Toxigenic	Both	*P*-value	No	Yes	*P*-value
**Equidae**										
**Necropsy year**										
2019	32	15	0.60	3	11	1	0.91	8	4	0.30
2020	28	8		3	5	0		5	0	
2021	13	4		1	3	0		3	0	
**Animal data**										
Sex										
Female	46	21	0.23	7	13	1	0.34	11	3	1.00
Male, gelding	26	6		0	6	0		5	1	
*Unknown^b^*	*1*	*0*								
Age										
Foal	33	15	0.55	6	9	0	0.42	6	3	0.79
Yearling	10	1		0	1	0		1	0	
Young	5	2		0	2	0		2	0	
Adult	25	9		1	7	1		7	1	
Breed										
Thoroughbred	30	12	0.80	6	6	0	0.15	6	0	**0.038**
Trotter	23	8		1	6	1		3	4	
Saddlebred horse	9	2		0	2	0		2	0	
Other breeds	9	5		0	5	0		5	0	
*Unknown^b^*	*2*	*0*								
Thoroughbred/all other breeds	30/41	12/15	1.0	6/1	6/13	0/1	**0.026**	6/10	0/4	0.27
Trotter/all other breeds	23/48	8/19	1.0	1/6	6/13	1/0	0.29	3/13	4/0	**0.007**
*Post-mortem* observations										
Signs of diarrhea										
One sample per animal: CAE	59	11	**0.0002**	3	8	0	1.0	8	0	0.12
Two samples per animal: CAE + CDL	14	16		4	11	1		8	4	
Signs of endo-enterotoxemia										
Yes	7	13	**0.00008**	3	9	1	1.0	7	3	0.58
No	65	14		4	10	0		9	1	
*Unknown^b^*	*1*	*0*								
Risk factors										
Previous antibiotic treatment										
Yes	24	11	0.48	5	6	0	0.18	3	3	**0.025**
No	43	14		2	11	1		12	0	
*Unknown^b^*	*6*	*2*		*0*	*2*	*0*		*1*	*1*	
Previous hospitalization										
Yes	22	11	0.47	4	7	0	0.65	7	0	0.25
No	49	16		3	12	1		9	4	
*Unknown^b^*	*2*	*0*		*3*	*12*	*1*		*9*	*4*	

^*a*^ This contingency table shows the composition of the equine population (number of necropsied animals). In lines, Equidae are categorized according to (i) necropsy year, (ii) identity data (sex, age, breed), (iii) *post*-*mortem* observations (signs of diarrhea or of endo-enterotoxaemia), (iv) *ante*-*mortem* data (previous antibiotic treatment or hospitalization) (Table S1). In columns, Equidae are categorized according to (i) *C. difficile* presence or not, assessed in the whole equine population (*n *= 100), (ii) *C. difficile* pathogenicity or not, in the subpopulation of *C. difficile*-positive animals (*n* = 27), and finally (iii) toxin production (virulence) or not, in the subpopulation of animals with a toxigenic *C. difficile* (*n* = 20).

^*b*^ The information was missing in the necropsy register (Table S1). In these cases, the number of animals is indicated in italics for full information, but these animals were excluded from the statistical analysis using Fisher’s exact test. Statistical significance: *P*-values < 0.05, which are shown in bold, on a grey background.

### *C. difficile* prevalence

Animal cecal contents (99 CAE) and watery intestinal contents (30 CDL) were tested for *C. difficile*, directly or after enrichment. After screening by standard PCRs (27, 28) and confirmation by MALDI-TOF, twenty-seven animals were positive for *C. difficile*, in their two samples in nine cases (Table S1). *C. difficile* presence in Equidae was independent of animal sex, age, breed, antibiotic treatment or hospitalization, but significantly correlated to signs of diarrhea or endo-enterotoxaemia (Table 1, Fig. S1).

### Pathogenicity

Thirty-four *C. difficile* isolates were preserved from twenty-five Equidae (Table 2). They form the first French library of *C. difficile* strains originating from Equidae, CloDifEqui (Table 2). Their pathogenicity profile was characterized by multiplex PCR, which revealed non-toxigenic isolates (*N* = 9) and toxigenic ones (*N* = 25) with three profiles (*tcdA^+^ tcdB^+^ cdt^+^*, *tcdA^+^ tcdB^+^* or *tcdB^+^*) (Table 2). The former isolates originated from seven animals, including six Thoroughbred, and the latter from nineteen animals of six breeds (Table S1). One animal (#29) was co-colonized by a non-toxigenic strain (CAE) and a toxigenic one (CDL). In the eight other animals with two isolates, both were either non-toxigenic (#18, #60) or toxigenic with the same profile (#3, #32, #36, #37, #42, #43) (Table 2).

**TABLE 2:** *C. difficile* strains isolated from necropsied Equidae^*a*^

*C. difficile*	Equidae		Multiplex PCR							Ribotype*^k^*	MLST^*l*^	Vancomycin	Metronidazole
Strain Name^*b*^	Identification Number	Digestive content	*tpi* ^*e*^	*tcdA* ^*f*^	*tcdB* ^*e*^	lok^*g*^	*tcdC*^*h*^ deletion	*cdt* ^*i*^	Profile^*j*^	(WEBRIBO)		MIC (mg/L)*^m^*	MIC (mg/L)^*m*^
2019-2021													
*Cd*E-3	3	CAE	+	+	+	−	−39	+	***tcdA^+^*** ***tcdB* ^+^** ***cdt^+^***	126	ST11	**1.0**	0.094
*Cd*E-3-CDL	3	CDL	+	+	+	−	−39	+		126	ST11	**1.0**	0.064
*Cd*E-19	19	CAE	+	+	+	−	−39	+		126	nd	0.38	0.047
*Cd*E-53	53	CAE^*d*^	+	+	+	−	−39	+		078	nd	0.50	0.094
*Cd*E-1	1	CAE	+	+	+	−	0	T	***tcdA^+^*** ***tcdB*** ^+^	AI-53	nd	0.25	0.125
*Cd*E-81-CDL	81	CDL^*d*^	+	+	+	−	0	T		FR227	nd	0.38	0.19
*Cd*E-42	42	CAE	+	+	+	−	0	T		020	nd	**0.75**	0.19
*Cd*E-42-CDL	42	CDL	+	+	+	−	0	T		020	nd	0.50	0.125
*Cd*E-43	43	CAE	+	+	+	−	0	T		020	ST2 ST2	0.50	0.19
*Cd*E-43-CDL	43	CDL	+	+	+	−	0	T		020		0.50	**0.25**
*Cd*E-57	57	CAE^*d*^	+	+	+	−	0	T		020	nd	0.50	0.125
*Cd*E-78	78	CAE^*d*^	+	+	+	−	0	T		020	nd	0.50	0.19
*Cd*E-9	9	CAE	+	+	+	−	0	T		012	ST54	0.50	0.047
*Cd*E-85	85	CAE	+	+	+	−	0	T		012	nd	0.50	0.047
*Cd*E-2	2	CAE	+	+	+	−	0	T		005	nd	**0.75**	0.125
*Cd*E-29-CDL^*c*^	29	CDL	+	+	+	−	0	T		005	nd	**0.75**	0.125
*Cd*E-90	90	CAE	+	+	+	−	0	T		005	nd	0.25	0.047
*Cd*E-12	12	CAE	+	del	+	−	0	−	***tcdB^+^***	017	ST37	0.50	0.094
*Cd*E-32	32	CAE	+	del	+	−	0	−		017	ST37	**0.75**	**0.38**
*Cd*E-32-CDL	32	CDL	+	del	+	−	0	−		017	ST37	**0.75**	0.19
*Cd*E-36	36	CAE	+	del	+	−	0	−		017	ST37	**0.75**	**0.25**
*Cd*E-36-CDL	36	CDL	+	del	+	−	0	−		017	ST37	**0.75**	0.19
*Cd*E-37	37	CAE	+	del	+	−	0	−		017	nd	**0.75**	**0.25**
*Cd*E-37-CDL	37	CDL	+	del	+	−	0	−		017	ST37	**0.75**	**0.38**
*Cd*E-92	92	CAE	+	del	+	−	0	−		017	nd	0.50	**0.25**
*Cd*E-29^*c*^	29	CAE	+	−	−	+	−	−	**Non- toxigenic**	439	nd	0.50	0.047
*Cd*E-91	91	CAE	+	−	−	+	−	−		035	nd	0.125	0.125
*Cd*E-5	5	CAE	+	−	−	+	−	−		009	ST3	0.50	0.094
*Cd*E-18	18	CAE	+	−	−	+	−	−		009	ST3	**0.75**	0.125
*Cd*E-18-CDL	18	CDL	+	−	−	+	−	−		009	ST3	0.50	0.094
*Cd*E-21	21	CAE	+	−	−	+	−	−		009	nd	0.125	0.094
*Cd*E-60	60	CAE	+	−	−	+	−	−		009	nd	**0.75**	0.094
*Cd*E-60-CDL	60	CDL	+	−	−	+	−	−		009	nd	0.50	0.064
*Cd*E-61-CDL	61	CDL^*d*^	+	−	−	+	−	−		009	nd	**0.75**	0.064
2018													
*Cd*E-0-CDL	0	CDL	+	del	+	−	0	−	***tcdB^+^***	017	nd	0.38	0.032

^*a*^ All preserved strains are listed. Their origin (animal and digestive content) is indicated. For each strain, molecular characterization results: multiplex PCR results, PCR-ribotype and Multi Locus Sequence Type (MLST, Table S3), are shown together with the Minimal Inhibitory Concentration (‘MIC’) of vancomycin and of metronidazole.

^*b*^ *Cd*E for *C. difficile* isolate originating from Equidae, followed by animal identification number, and possibly by ‘‘CDL’’ when the isolate was recovered from a watery content.

^*c*^ The two strains co-colonizing the same animal.

^*d*^ The single digestive content (among the two ones recovered from the animal) from which *C. difficile* was isolated.

^*e*^ PCR-fragment of wild-type size (“+”) or absence of amplification (“−”).

^*f*^ PCR-fragments of wild-type size (“+”) or of shorter size indicative of a deletion (“del”) or absence of amplification (“−”).

^*g*^ PCR-fragment of 117 bp (“+,” i.e., Pathogenicity Locus deletion and non-toxigenic strain) or no amplification (“−”).

^*h*^ 39bp-deletion (“-39”) or absence of deletion (“0,” i.e., PCR-fragment of wild-type size) or absence of amplification (“−”).

^*i*^ PCR-fragment of wild-type size (“+”) or truncated PCR-fragment (“T,” i.e., presence of a pseudogene) or absence of amplification (“−”).

^*j*^ Toxin gene profile according to multiplex PCR results: toxin genes of wild-type size or “non- toxigenic”.

^*k*^ PCR-ribotype assigned according to the WEBRIBO database.

^*l*^ MLST: Multi-Locus Sequence Type; nd, not determined.

^*m*^ MIC, Minimum inhibitory concentration; the two highest values are in bold.

### Ribotype diversity

CloDifEqui isolates were assigned to 11 ribotypes and 4 clades (1, 2, 4, and 5) (Table 2; Table S2) (30). Toxigenic isolates were of eight ribotypes: 126 and 078 (*tcdA^+^ tcdB^+^ cdt^+^* and the same *tcdC* truncation), 020, 005, 012, AI-53, and FR227 (*tcdA^+^ tcdB*^+^), and 017 (*tcdB^+^*). Non-toxigenic isolates belonged to ribotypes 009, 035, and 439. The two most prevalent ribotypes (*n* = 5 animals) were 017 (*N* = 8 isolates) and 009 (*N* = 7).

The two strains of animal #29, which were toxigenic and non-toxigenic, belonged to ribotype 005 and 439. In all other pairs of isolates originating from the same animal, both were of the same ribotype (Table 2).

### Susceptibility to metronidazole and to vancomycin

All strains were susceptible to the antibiotics used to treat CDI in Equidae, metronidazole and vancomycin (MICs lower than EUCAST breakpoints: 2 mg/L in both cases; https://www.eucast.org/fileadmin/src/media/PDFs/EUCAST_files/Breakpoint_tables/v_15.0_Breakpoint_Tables.pdf) (Table 2). For metronidazole, strain MICs ranged from 0.032 to 0.38 mg/L, with a median value of 0.13 mg/L. For vancomycin, strain MICs ranged from 0.13 to 1 mg/L, with a median value of 0.5 mg/L. Strains belonging to ribotypes 017 and 126 showed the highest MIC for metronidazole (0.38 mg/L) and for vancomycin (1.0 mg/L), respectively.

### Co-location of Equidae sharing the same ribotype

**Fig 1:**
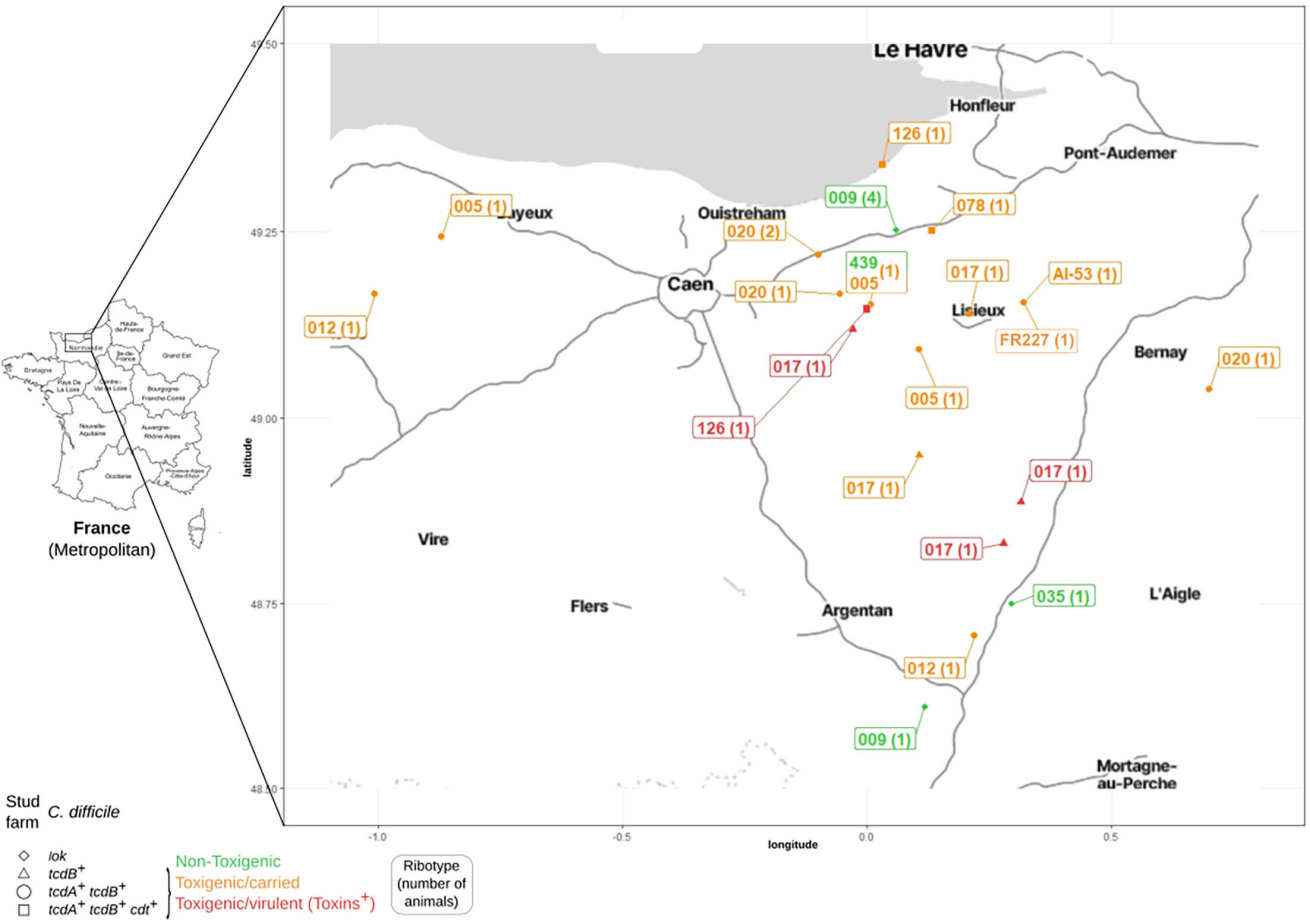
Geographical location of *C. difficile*-positive Equidae. All *C. difficile*-positive Equidae are shown on a map of Normandy according to the position of their stud farm (Table S1). The premises of each animal is indicated by a symbol depending on *C. difficile* toxin gene profile (multiplex PCR): squares for *tcdA^+^ tcdB^+^ cdt^+^*, circles for *tcdA^+^ tcdB*^+^, triangles for *tcdB^+^,* and diamonds for non-toxigenic strains (Table 2). *C. difficile* ribotype (Table 2) is indicated in a box, and when several animals harbored this ribotype in the same premises (Table S1), their number appears between brackets. *C. difficile* pathogenicity and virulence are represented using a color code: (i) green for non-toxigenic, (ii) orange for toxigenic and carried, and (iii) red for toxigenic and virulent (having produced toxins). The map was generated in RStudio using the ggmap package, using underlying geographic data from OpenStreetMap.

We cross-analyzed Equidae location and *C. difficile* ribotypes (Fig. 1; Table S1). In two premises between Caen and Pont-Audemer, some animals shared the same ribotype: (i) 020, for two co-located Equidae necropsied in 2019 (#42 and #43) and (ii) 009 for four co-located Equidae necropsied in 2019 (#5, #18, and #21) and 2020 (#61). This suggested (i) that in each premises, isolates of the same ribotype might be clonal and (ii) that transmission between animals or from a common environmental source might have occurred.

### Toxin production

We tested the presence of free toxins in equine digestive contents using a standard enzymatic immuno-assay, initially developed for human fecal materials, but also used in animals, notably foals (34). To check the assay specificity when applied to equine digestive contents, we included samples expected to be toxin-negative: (i) all 10 samples displaying a non-toxigenic *C. difficile* (Table 3) and (ii) 27 *C. difficile*-negative samples (15 CDL and all samples from animals with endo-enterotoxaemia signs) (Table 3; Table S1 and Table S3). No toxins could be detected in any of them, while GDH was positive in seven of the former (Table 3; Table S3).

**TABLE 3:** Free toxin detection^*a*^

Equidae number	Digestive content	Strain^*b*^	Enrichment^*c*^	Pathogenicity^*d*^	Ribotype	GDH^*e*^	Free toxins^*e*^ Toxins A and B
**3**	CAE	*Cd*E-3	−	**Toxigenic**	126	+	**+**
	**CDL**	*Cd*E-3-CDL	−	**Toxigenic**	126	+	**+**
**32**	CAE	*Cd*E-32	−	**Toxigenic**	017	+	**+** (weak)
	**CDL**	*Cd*E-32-CDL	−	**Toxigenic**	017	+	**+** (weak)
**36**	CAE	*Cd*E-36	−	**Toxigenic**	017	+	**+**
	**CDL**	*Cd*E-36-CDL	−	**Toxigenic**	017	+	**+**
**37**	CAE	*Cd*E-37	−	**Toxigenic**	017	+	**+**
	**CDL**	*Cd*E-37-CDL	−	**Toxigenic**	017	+	**+**
29^*f*^	CAE	*Cd*E-29	+	Non-Toxigenic	439	+ (weak)	−
	**CDL**	*Cd*E-29-CDL	−	**Toxigenic**	005	+ (weak)	−
42	CAE	*Cd*E-42	+	**Toxigenic**	020	−	−
	**CDL**	*Cd*E-42-CDL	+	**Toxigenic**	020	−	−
43	CAE	*Cd*E-43	−	**Toxigenic**	020	+	−
	**CDL**	*Cd*E-43-CDL	−	**Toxigenic**	020	+	−
53	CAE	*Cd*E-53	+	**Toxigenic**	078	−	−
	**CDL**	None	NA	NA	NA	−	−
54	CAE	None	NA	NA	NA	−	−
	**CDL**	Not preserved	−	**Toxigenic**	ND	−	−
57	CAE	*Cd*E-57	−	**Toxigenic**	020	−	−
	**CDL**	None	NA	NA	NA	−	−
78	CAE	*Cd*E-78	+	**Toxigenic**	020	−	−
	**CDL**	None	NA	NA	NA	−	−
81	CAE	None	NA	NA	NA	−	−
	**CDL**	*Cd*E-81-CDL	+	**Toxigenic**	FR227	−	−
1	CAE	*Cd*E-1	+	**Toxigenic**	AI-53	−	−
2	CAE	*Cd*E-2	+	**Toxigenic**	005	+ (weak)	−
9	CAE	*Cd*E-9	−	**Toxigenic**	012	+	−
12	CAE	*Cd*E-12	−	**Toxigenic**	017	−	−
19	CAE	*Cd*E-19	−	**Toxigenic**	126	−	−
85	CAE	*Cd*E-85	−	**Toxigenic**	012	+	−
90	CAE	*Cd*E-90	+	**Toxigenic**	005	−	−
92	CAE	*Cd*E-92	−	**Toxigenic**	017	−	+ (weak)
18	CAE	*Cd*E-18	−	Non-toxigenic	009	+	−
	**CDL**	*Cd*E-18-CDL	−	Non-toxigenic	009	+	−
48	CAE	None	NA	NA	NA	−	−
	**CDL**	Not preserved	−	Non-toxigenic	ND	−	−
60	CAE	*Cd*E-60	−	Non-toxigenic	009	+	−
	**CDL**	*Cd*E-60-CDL	−	Non-toxigenic	009	+	−
61	CAE	None	NA	NA	NA	−	−
	**CDL**	*Cd*E-61-CDL	+	Non-toxigenic	009	+	−
5	CAE	*Cd*E-5	−	Non-toxigenic	009	+	−
21	CAE	*Cd*E-21	−	Non-toxigenic	009	−	−
91	CAE	*Cd*E-91	−	Non-toxigenic	035	+	−

^*a*^ The results of free toxin detection are shown for the digestive contents of all *C. difficile*-positive Equidae. For each Equidae, identification number and recovered digestive content(s) (CAE and possibly CDL, in bold) are indicated. For each digestive content, *C. difficile* strain and pathogenicity are shown together with the results of toxin and GDH detection (enzymatic immuno-assay). Animals are listed according to the pathogenicity of their *C. difficile* isolate(s) and to their *post-mortem* observed signs of diarrhea (according to their recovered contents: both CAE and CDL, or CAE alone).

^*b*^ Strain name or “not preserved” (an initially detected strain that could not be recovered later) or “none” (absence of *C. difficile* detection).

^*c*^ Method used to isolate the strain: Enrichment (“+”) or not (“−,” i.e., direct inoculation on plates).

^*d*^ Toxigenic (in bold) or non-toxigenic strain.

^*e*^ Detection (“+”) or absence of detection (“−”); “weak” was indicated for a detected band of lower intensity than that of the positive control, and such a weak positive result was confirmed by two independent observations. NA, not applicable; ND, not determined.

^*f*^ Co-colonized animal.

**Fig 2:**
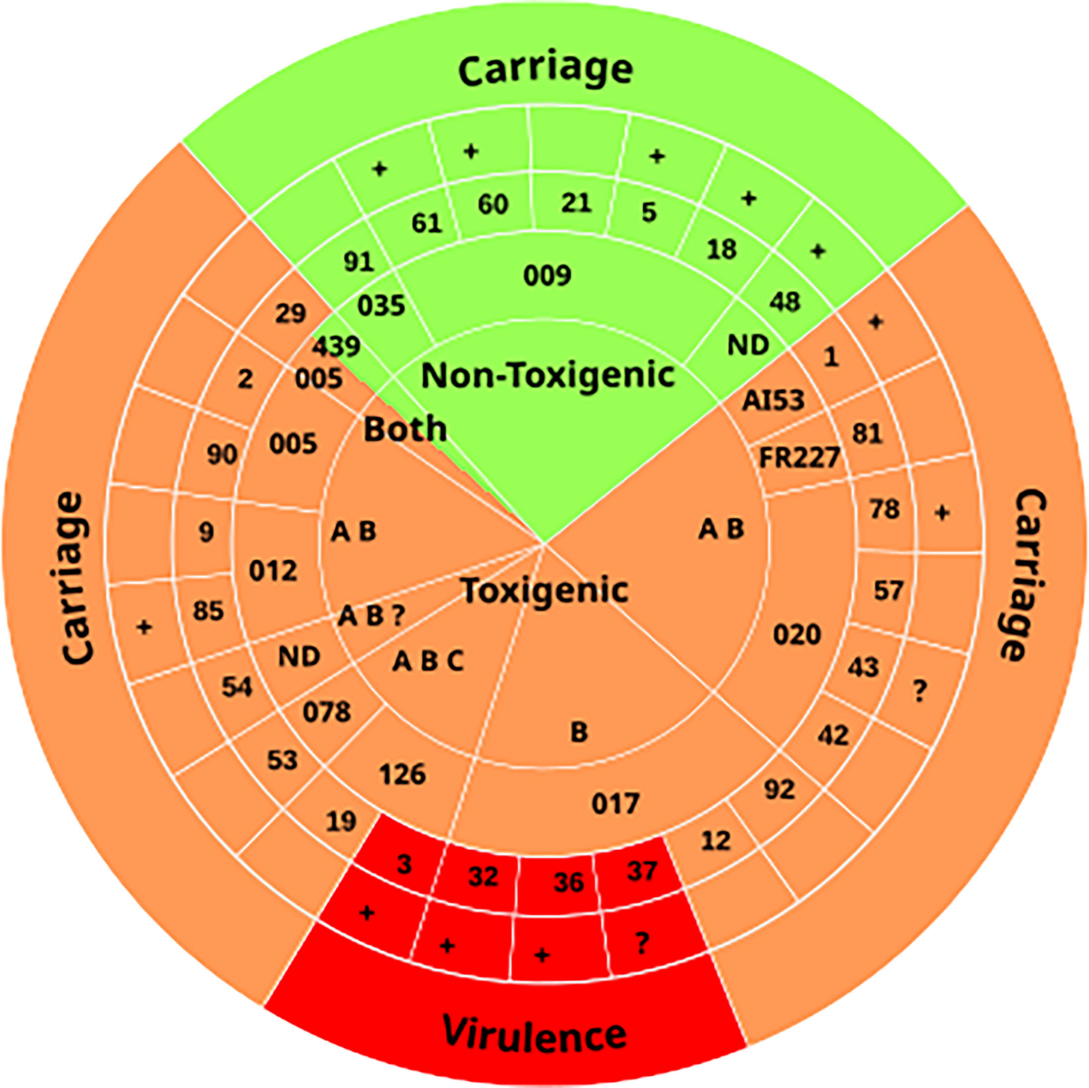
*C. difficile* virulence or carriage in *C. difficile*-positive Equidae. All *C. difficile*-positive animals are shown as a wedge of a pie chart. For each positive animal, the pathogenicity, ribotype, and virulence of *C. difficile* are indicated in four concentric rings. First (core): *C. difficile* pathogenicity (Table 2). Non-toxigenic and toxigenic strains are indicated on a green or orange background, respectively. “Both” is indicated when a non-toxigenic strain and a toxigenic strain were co-colonizing the same animal (#29, bi-colored wedge). The toxin gene profile of toxigenic strains is specified using the following abbreviations: A for *tcdA^+^*, B for *tcdB^+^*, C for *cdt^+^* and “?” when the presence of *cdt* genes has not been tested. Second: *C. difficile* ribotype (according to the WEBRIBO database) (Table 2). Third: Animal identification number. Fourth: Antibiotic treatment in the 2–3 weeks before death (“+”) or not. Fifth (outer ring): Virulence (Toxin production, Table 3) or carriage. Virulence, carriage of a toxigenic strain, and carriage of a non-toxigenic strain are on a red, orange, and green background, respectively.

Free toxins and GDH were detected in the two samples of four animals (#3, #32, #36, and #37), indicating *C. difficile* virulence (Table 3). These animals displayed signs of diarrhea (CDL in the colon) and a toxigenic *C. difficile* (ribotype 126 or 017) was identified as the only enteropathogen (Table 3, Fig. 2). These data were highly suggestive of animal CDI. The 16 remaining Equidae with a toxigenic *C. difficile*, in which no toxin was detected (Table 3), were carriers. Half of them showed no sign of diarrhea (#1, #2, #9, #12, #19, #85, #90, and #92), suggesting asymptomatic carriage. For almost all others (except #57), signs of diarrhea might be due to another identified intestinal pathogen (*C. perfringens* in #43, *C. perfringens* or *P. sordellii* in #29 and #42, *C. perfringens*, *P. sordellii* or a rotavirus in #53, and a parasite in #78) or to the non-infectious intestinal disease having caused death (torsion of the large colon in #54 and intestinal lymphoma in #81) (Table S1).

Among Equidae with a toxigenic *C. difficile*, we compared those in which toxins were detected to the others. These two subpopulations were similar in terms of sex, age, and non-pathognomonic signs of endo-enterotoxaemia or diarrhea, but they significantly differed in terms of breed and antibiotic treatment (Table 1). Three out of 4 toxin-positive animals (all Trotters) compared to 3 out of 16 carriers of a toxigenic *C. difficile* had received antibiotics (Table 1, Fig. 2; Table S1). In the subpopulation with a toxigenic *C. difficile*, toxins were, therefore, produced in half of the antibiotic-treated animals (3/6), but in none of the untreated ones (0/12) (Table 1, Fig. 2). Antibiotic treatments could, therefore, have increased the risk for Equidae to develop CDI.

### *C. difficile* as the most probable cause of animal death

We examined whether *C. difficile* could have caused the death of the four toxin-positive animals, by analyzing available data (Table S1). The fulminant death of animal #3, a 15-day-old foal, was not primarily due to *C. difficile* but to small intestine intussusception (Table S1). Animal #37, an adult, died from multiple infections (respiratory, musculoskeletal and digestive infections, including endo-enterotoxaemia), probably due to *Klebsiella pneumoniae*, which was identified in lungs, lung abscess and hock muscle, and/or *C. difficile* (Table S1).

For the last two animals, #32 and #36, both foals, death was due to endo-enterotoxemia (Table S1 and Fig. S2). *C. difficile* was the only identified enteropathogen (Table S1) and considering the presence of toxins (Table 3), the most probable cause of death. The resulting prevalence of *C. difficile* as the cause of animal death (2%) was of the same range as the previously measured ones (0%–5%). Of note, ribotype 017 was implicated in both cases and likely also in one case of the 2018 pilot study: animal #0, which died from endo-enterotoxaemia following antibiotic treatment, displayed signs of diarrhea and a *C. difficile* of ribotype 017 as the only enteropathogen (Table 2; Table S1).

### Discussion

### *C. difficile* prevalence, diversity, and epidemiology

Here, we evaluated the presence of *C. difficile* in Equidae necropsied in France for the first time. *C. difficile* prevalence in these Equidae having died from diverse, mainly non-intestinal diseases (Table S1), was 27%. It was similar to, and sometimes lower than, the prevalence reported in live horses in Europe (4%–33% for healthy animals and 5%–63% for sick animals with intestinal illnesses) (10) or in Australia (31% for animals with diarrhea or not) (35). The prevalence of toxigenic strains was also similar in French Equidae (20%) and Australian horses (18%) (35).

*C. difficile* strains from Equidae belonged to eleven already described ribotypes (10, 36). Toxigenic strains were of eight ribotypes. The historical epidemic ribotype 027 (1), described in horses (20), was not identified here, probably because of the changing *C. difficile* epidemiology and 027 prevalence decrease (36–38). Most ribotypes of CloDifEqui toxigenic strains are common in humans: ribotypes 020, 078, 126, and 005 were in the Top 10 ribotypes in European patients in 2013–2014, and ribotypes 017 and 012 in the Top 20 (36). In Czech horses (*n* > 200), toxigenic strains were of only five ribotypes (24), including one (012) in the Top 20 (36). In horses in Australia (*n* ~ 400), toxigenic strains belonged to 40 ribotypes, including 18 internationally known ribotypes like 012, 014/020, and 087, locally identified and novel ribotypes (35). Finally, our study revealed that French Equidae harbored diverse, already described, and mostly clinically important ribotypes.

Most ribotypes of CloDifEqui toxigenic strains are also common in animals (10, 12). Ribotypes 126, 020, and 012 had been found in farm animals (10). Ribotype 078, known as prevalent in animals, notably pigs (10), was relatively uncommon here (*N* = 1 isolate), but the phylogenetically close ribotype 126 was represented (*N* = 3) (Table 2) (39). Of note, the ribotypes of most CloDifEqui toxigenic strains have been detected in very diverse sources in Europe, including mollusks (005, 012, 017, 020, 078, 126, and AI-53) (40), potatoes, and vegetables (012, 020, 078, and 126) (12, 41).

The diversity and ubiquity of ribotypes identified here supported a role for Equidae in the cycle of *C. difficile* transmission in France, which is important in a One Health perspective (11, 20). Many different environmental, animal or human sources could have contaminated French Equidae during their life. In turn, they could have contributed to *C. difficile* dissemination and transmission to other hosts of the same or other species including humans. The cross-analysis of Equidae location and *C. difficile* ribotypes revealed that in two cases, animals of the same premises displayed the same ribotype, indicating strain phylogenetic proximity and raising the possibility of a single clonal lineage. Each of the two premises might represent a cluster of contamination and of transmission between Equidae, either directly or *via* a common source. Genome-wide investigations are under progress to analyze the phylogeny of *C. difficile* isolates originating from Equidae (CloDifEqui collection) and from patients of the same region during the same period. This should help understand *C. difficile* circulation in Normandy and evaluate zoonotic or anthropo-zoonotic transmission, an important One Health issue.

### Toxin production

After PCR amplification of toxin genes, a method of high sensitivity and negative prediction value, we performed toxin detection, a method of high specificity and positive prediction value, as recommended for CDI diagnosis in European patients (4), but rarely performed in animals including Equidae (42–45). Four animals with both signs of diarrhea and a toxigenic *C. difficile* were positive for toxins, suggesting *in vivo* (*ante-mortem*) virulence and CDI in 4% of Equidae. Carriers of a toxigenic *C. difficile* represented 16% of the population (Table 3, Fig. 2), a high and previously overlooked level.

In Equidae, *C. difficile* presence (Table 1; Table S1) was significantly correlated to animal signs of diarrhea or endo-enterotoxemia. In *C. difficile* carriers, in the absence of toxins, these signs were unexpectedly more frequent than in negative animals (12/23 vs 15/75). However, the former, compared to the latter, also more frequently harbored another enteropathogen (bacterium, virus, parasite) (7/23 vs 9/73) (Table S1), which might be responsible for diarrhea. The most prevalent was *C. perfringens* in four animals, three of which also harbored *P. sordelli* (Table S1). Two carriers of a toxigenic *C. difficile* died from endo-enterotoxaemia, which might have been caused by (i) *C. perfringens* (#43) or (ii) *C. perfringens* and/or *P. sordellii* (#42) (Table S1 and Fig. S2). Host co-colonization or co-infection by *C. difficile* and *C. perfringens* have been described in horses (46, 47), dogs (48), pigs (49), and humans (50).

In this study, the most complex case was animal #29, which displayed three clostridial enteropathogens and two *C. difficile* strains, one toxigenic and the other non-toxigenic (Table S1; Table 2). Co-colonization by a toxigenic strain and a non-toxigenic one, notably of ribotype 009, has been reported in companion animals (51). Non-toxigenic strains are able to prevent CDI or reduce its severity in animal models (52) and a ribotype 009 strain can protect both model and commercial piglets (49). Whether CloDifEqui non-toxigenic strains, especially of ribotype 009, could provide protection against a toxigenic strain would require further investigations.

*C. difficile* had produced toxins in four Trotters and at least three had received antibiotics (Table 1; Table S1). Of note, among the eight *C. difficile* carriers having received antibiotics, five displayed non-toxigenic strains unable to cause disease (Fig. 2). Antibiotic treatments are known to favor microbiota dysbiosis, *C. difficile* outgrowth and ultimately, virulence (2). Antibiotic treatments alter the metabolic functions of the microbiota, resulting in the loss of colonization resistance by several mechanisms. One of them is the accumulation of primary bile salts, which favor spore germination and do not inhibit vegetative multiplication (53).

The *C. difficile* strains that had produced toxins in Equidae were of ribotypes 126 and 017. Ribotype 126 was the sixth most prevalent ribotype in European patients in 2013–2014 (36). It is also, together with its close relative 078, prevalent in production animals, notably pigs (10, 54), and in biogas plants using animal manure in another French region, Brittany (55). Ribotype 017 is prevalent in patients in Asia (56), common in Europe (36), notably in Greece (2), but less frequent in France, where it was undetected in 2022 (36, 38). Ribotype 017 is also described as relatively uncommon in animals, notably compared to ribotype 078 (10, 56). For example, in Japan, in 34 horses with CDI, ribotype 017 was clearly less prevalent than ribotype 078 (< 10% vs 35%) (57). As ribotype 017 was not detected in biogas plants in Brittany (55), its prevalence in French Equidae (5% of the whole population and 25% of the subpopulation with a toxigenic *C. difficile*) was noticeable. Moreover, its virulence in three close animals (Fig. 1) within a short period (Table S1) raised the possibility of a local infection cluster.

In conclusion, CDI likely affected 4% of the French Equidae under study before their death, in most cases after an antibiotic treatment. Ribotype 017, which was unexpectedly the most prevalent ribotype, was also the most virulent and should be carefully monitored in the future. Moreover, as many as 20% of animals were able to disseminate toxigenic *C. difficile.* French Equidae, therefore, represented an underestimated reservoir of clinically important *C. difficile*, with a risk of zoonotic transmission and contribution to community-acquired infections. Our findings highlight the importance of a One Health perspective in the epidemiological surveillance of *C. difficile*.

## Data Availability

All data are available in the manuscript.

## SUPPLEMENTAL MATERIAL

Figure S1 spectrum.02165-25-s0001.tif Pie charts.

Figure S2 spectrum.02165-25-s0002.tif Cause of death.

Table S1 spectrum.02165-25-s0003.pdf Animal data.

Table S2 spectrum.02165-25-s0004.xlsx Sequence types (MLST) of CloDifEqui strains.

Table S3 spectrum.02165-25-s0005.xlsx Toxin detection in *C. difficile*-negative animals.

ASM does not own the copyrights to Supplemental Material that may be linked to, or accessed through, an article. The authors have granted ASM a non-exclusive, world-wide license to publish the Supplemental Material files. Please contact the corresponding author directly for reuse.
