# Supplementary figures and images for: Clostridioides difficile in equidae necropsied in Northwestern France, between 2019 and 2021
Source: Microbiol Spectr. 2025 Dec 30;14(2):e02165-25. doi: 10.1128/spectrum.02165-25 (PMC12889072; doi:10.1128/spectrum.02165-25)

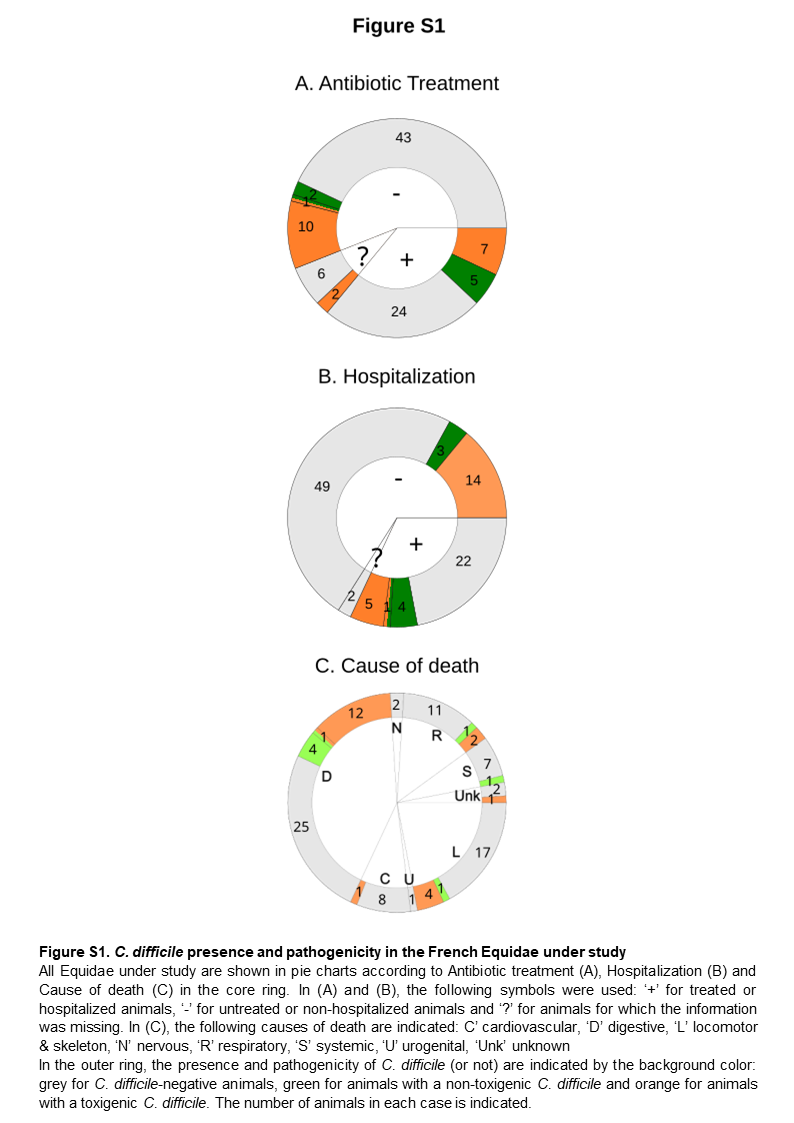

Supplement: Figure S1 — Pie charts. [file spectrum.02165-25-s0001.tif]

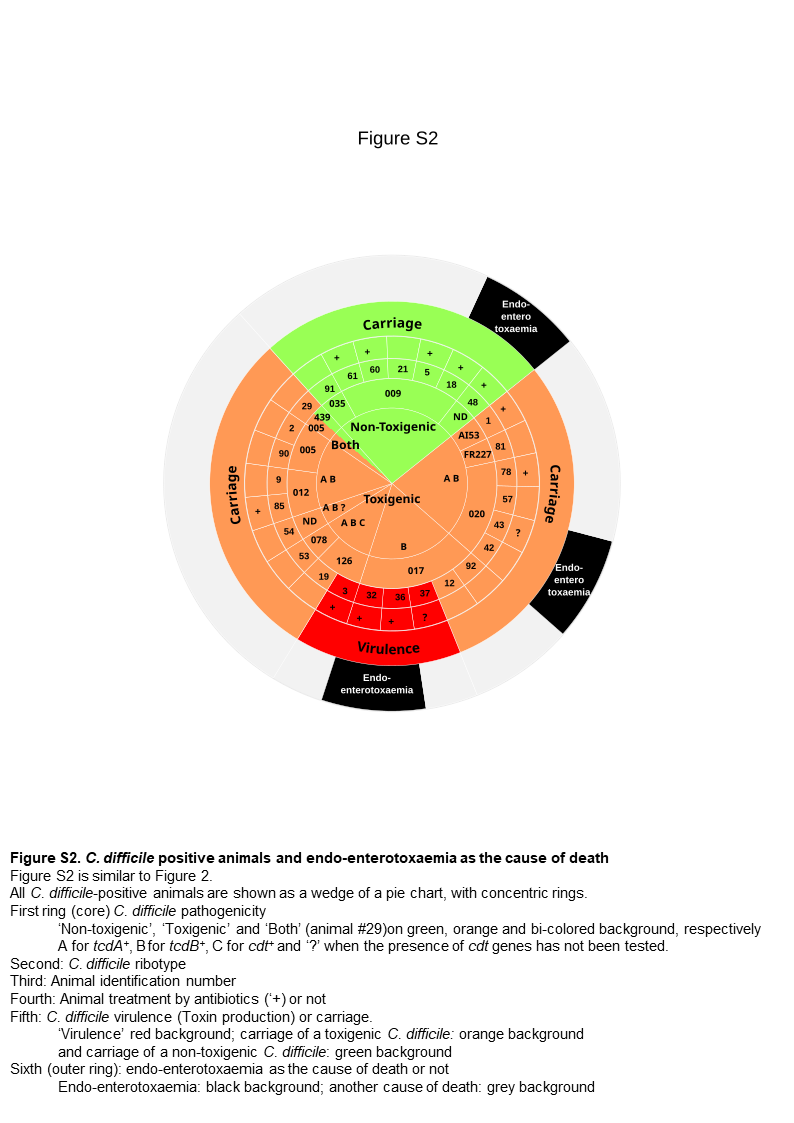

Supplement: Figure S2 — Cause of death. [file spectrum.02165-25-s0002.tif]
