# Supplementary material for: Clostridioides difficile in equidae necropsied in Northwestern France, between 2019 and 2021
Source: Microbiol Spectr. 2025 Dec 30;14(2):e02165-25. doi: 10.1128/spectrum.02165-25 (PMC12889072; doi:10.1128/spectrum.02165-25)
Supplement: Table S1 — Animal data. [file spectrum.02165-25-s0003.pdf]

Supplementary Table 1: Animal Data

	Usual data registered after necropsy														Data specific from this study	
	Necropsy date		Animal data			Animal/premises location			*Ante-mortem* data			*Post-mortem* data	Cause of death			
**Animal Identi-fication**	**Year**	**Month**	**Sex**	**Age range**	**Breed**	**French region**	**Zip code**	**Same pre-mises**	**Antibiotic treatment**	**Hospita-lisation**	**Suspicion of endo-entero toxæmia**	**Standard microbial or molecular analyses** in relevant samples to identify pathogens in case of *post-mortem* observed signs of infection	**Cause of death**	**Type of disease** (tract/system)	**Recovered digestive contents**	***C. difficile*** **absence (-) or presence (+) and pathogenicity**
**Systematic study 2019-2021**																
**1**	2019	May	Male	Foal	Irish Cob	Normandy	14100	§	Yes	No	No	Lung, Liver, kidney: non-hemolytic *E. coli* and *Enterococcus* spp.	nutritional myopathy (white muscle disease)	locomotor & skeleton	Caecum	+ toxigenic (*tcdA*$^+$ *tcdB*$^+$)
**2**	2019	May	Gelding	Adult	Lusitano	Normandy	14330		No	No	No	No bacteriological analysis	Anoxia/hypoxia, not pathognomonic	respiratory	Caecum	+ toxigenic (*tcdA*$^+$ *tcdB*$^+$)
**3**	2019	May	Male	Foal	French Trotter	Normandy	14340		Yes	Yes	No	Caecum, ascending colon, small intestine: non-hemolytic *E. coli, Enterococcus faecalis*	Small intestinal intussusception	digestive	Caecum, descending & ascending colons	+ toxigenic (*tcdA*$^+$ *tcdB*$^+$ *cdt*$^+$)
**4**	2019	May	Female	Foal	Thoroughbred	Normandy	61310		No	No	No	Placenta, Lung, Liver, kidney: *Pasteurella pneumotropica,* non-hemolytic *E. coli* , *Enterococcus* spp.	*Pasteurella pneumotropica* infection	systemic	Caecum	-
**5**	2019	May	Female	Foal	Thoroughbred	Normandy	14430	*	Yes	No	No	Lung, lung abscess: *Rhodococcus equi, Enterococcus* spp.	Interstitial pneumonia	respiratory	Caecum	+ non-toxigenic
**6**	2019	May	Male	Foal	Thoroughbred	Normandy	14270		Yes	Yes	No	Lung, liver, kidney: *Enterococcus* spp. in addition to non-hemolytic *E. coli* in lung; small intestine content: non-hemolytic *E. coli, Enterococcus faecalis, Staphylococcus xylosus*	Not pathognomonic	unknown	Caecum	-
**7**	2019	May	Female	Adult	French Trotter	Normandy	61240		No	No	No	No bacteriological analysis	Multiparcellar fracture of the basal cranial bones	locomotor & skeleton	Caecum	-
**8**	2019	May	Female	Foal	Irish Cob	Normandy	14100	§	No	No	No	Placenta, lung, Liver, kidney: *Aeromonas salmonicida, Enterococcus* spp., non-hemolytic *E. coli* in addition to group I *Bacillus* spp., *Staphylococcus xylosus* in placenta; small intestine content: non-hemolytic *E. coli, Enterococcus faecalis, Aeromonas salmonicida, Staphylococcus xylosus*	Systemic infection, nutritional myopathy (white muscle disease)	locomotor & skeleton	Caecum	-
**9**	2019	May	Female	Foal	Thoroughbred	Normandy	61240		No	No	No	Lung, Liver, kidney: non-hemolytic *E. coli* and *Enterococcus* spp.	Not pathognomonic	unknown	Caecum	+ toxigenic (*tcdA*$^+$ *tcdB*$^+$)
**10**	2019	May	Male	Foal	Thoroughbred	Normandy	14140	!	Yes	Yes	No	Lung: non-hemolytic *E. coli*	Interstitial pneumonia	respiratory	Caecum	-
**11**	2019	June	Female	Adult	Trotter	Normandy	14140		No	Yes	No	No bacteriological analysis	Potential upper cervical trauma	locomotor & skeleton	Caecum	-
**12**	2019	June	Female	Adult	Thoroughbred	Normandy	14140		No	No	No	No bacteriological analysis	Multiparcellar fracture of the left tibi	locomotor & skeleton	Caecum	+ toxigenic (*tcdB*$^+$)
**13**	2019	June	Female	Adult	French Trotter	Normandy	61230		No	No	No	No bacteriological analysis	Torsion of the ascending colon	digestive	Caecum, Ascending colon	-
**14**	2019	June	Gelding	Yearling	Thoroughbred	Normandy	14140	ù	No	Yes	No	No bacteriological analysis	Potential upper cervical trauma	locomotor & skeleton	Caecum	-
**15**	2019	June	Female	Adult	French Trotter	Normandy	61150		No	No	No	No bacteriological analysis	compressive cervical myelopathy	locomotor & skeleton	Caecum	-
**16**	2019	June	Female	Yearling	Arabian horse	Normandy	61240	}	Yes	No	No	Small intestine content: non-hemolytic *E. coli, Enterococcus faecalis, Proteus mirabilis* ; caecal wall, caecal lymphatic node: *Rhodococcus equi* , non-hemolytic *E. coli, Enterococcus* spp.	*Rhodococcus equi* infection	digestive	Caecum, ascending colon	-
**17**	2019	June	Female	Yearling	French Trotter	Normandy	61100		No	No	No	No bacteriological analysis	Distension of digestive tract	digestive	Caecum	-
**18**	2019	June	Female	Foal	Thoroughbred	Normandy	14430	*	Yes	No	Yes	Lung, caudal lobes: non-hemolytic *E. coli, Enterococcus* spp., *Rhodococcus equi* ; pool of digestive contents: non-hemolytic *E. coli, Enterococcus faecalis*	*Rhodococcus equi* infection & endo-enterotoxemia	digestive	Caecum, descending & ascending colons	+ non-toxigenic
**19**	2019	June	Female	Young	Thoroughbred	Normandy	14910		No	No	Yes	Small intestine content: non-hemolytic *E. coli, Enterococcus faecalis*	Severe hemorrhagic enteritis	digestive	Caecum	+ toxigenic (*tcdA*$^+$ *tcdB*$^+$ *cdt*$^+$)

**20**	2019	June	Male	Foal	French Saddle	Normandy	50680	µ	No	No	Yes	Lung, Liver, kidney: *Salmonella* spp, *Streptococcus zooepidemicus* , non-hemolytic *E. coli, Enterococcus* spp.; caecum, ascending colon, small intestine: *Salmonella* spp, non-hemolytic *E. coli, Enterococcus faecalis*	Endo-enterotoxemia	digestive	Caecum, descending & ascending colons	-
**21**	2019	June	Female	Foal	Thoroughbred	Normandy	14430	*	No	Yes	No	Lung: *Streptococcus zooepidemicus, Enterococcus* spp.; stifles: *Streptococcus zooepidemicus, Enterococcus* spp., non-hemolytic *E. coli, Staphylococcus xylosus*	Severe septic arthritis of the stifle and pneumonia	locomotor & skeleton	Caecum	+ non-toxigenic
**22**	2019	June	Male	Foal	French Saddle	Normandy	61160		No	No	No	No bacteriological analysis	Small intestinal volvulus	digestive	Caecum	-
**23**	2019	June	Female	Adult	French Trotter	Normandy	14430	^	Unknown	No	No	Liver, liver abscesses: non-hemolytic *E. coli, Streptococcus equi, Enterococcus* spp.	Hepatopathy	digestive	Caecum	-
**24**	2019	July	Female	Foal	Thoroughbred	Normandy	14340		Yes	Yes	Yes	Lung: *Streptococcus zooepidemicus,* non-hemolytic *E. coli, Enterococcus* spp.; pool of digestive contents: *Streptococcus zooepidemicus* , non-hemolytic *E. coli, Enterococcus faecalis*	Endo-enterotoxemia & acute bronchopneumonia	respiratory	Caecum, descending & ascending colons	-
**25**	2019	July	Female	Adult	Thoroughbred	Normandy	27800		No	Yes	No	Guttural pouchs: *Actinobacillus equili, Streptococcus zooepidemicus, Enterococcus* spp., *Aspergillus*	Guttural pouch mycosis	respiratory	Caecum	-
**26**	2019	July	Female	Adult	Saddlebred hc	Normandy	50240		No	Yes	No	No bacteriological analysis	Perforation of the descending colon associated with sero-congestive peritonitis	digestive	Caecum	-
**27**	2019	July	Female	Foal	Arabo-Friesiar	Centre-Val I	36290		Yes	No	No	Lung, Liver, kidney: *Enterococcus* spp.	Coagulation deficiency	cardiovascular	Caecum	-
**28**	2019	July	Male	Foal	French Saddle	Normandy	50680	µ	Yes	No	No	No bacteriological analysis	Myopathy	locomotor & skeleton	Caecum	-
**29**	2019	July	Female	Adult	French Trotter	Normandy	14340		No	Yes	Yes	Intestinal content: non-hemolytic *E. coli, C. difficile, Clostridium perfringens, Paeniclostridium sordelli*	Enteritis associated to distension of the digestive tract	digestive	Caecum, Intestine	+ both non-toxigenic & toxigenic (*tcdA*$^+$ *tcdB*$^+$), co-colonisation
**30**	2019	July	Male	Foal	Thoroughbred	Normandy	14430	*	Yes	Yes	No	Lung: non-hemolytic *E. coli, Enterococcus* spp.; lung abscess: *Rhodococcus equi, Enterococcus* spp.	Bronchiolo-interstitial pneumonia	respiratory	Caecum	-
**31**	2019	July	Female	Adult	French Trotter	Normandy	61500	$	No	No	No	No bacteriological analysis	Rupture of the spleen	cardiovascular	Caecum	-
**32**	2019	July	Female	Foal	French Trotter	Normandy	61470		Yes	No	Yes	Caecum: non-hemolytic *E. coli, C. difficile*	Endo-enterotoxemia	digestive	Caecum, ascending colon	+ toxigenic (*tcdB*$^+$)
**33**	2019	July	Male	Foal	Thoroughbred	Normandy	61500	$	Yes	No	No	Lung: non-hemolytic *E. coli* ; lung abscess: *Rhodococcus equi*	*Rhodococcus equi* infection	respiratory	Caecum	-
**34**	2019	July	Male	Foal	Thoroughbred	Normandy	14160		No	Yes	No	No bacteriological analysis	Cervical trauma	locomotor & skeleton	Caecum	-
**35**	2019	July	Female	Adult	French Trotter	Normandy	61310		No	No	No	No bacteriological analysis	Torsion of ascending colon	digestive	Caecum, ascending colon	-
**36**	2019	August	Female	Foal	French Trotter	Normandy	61230	~	Yes	No	Yes	Pool of digestive contents: non-hemolytic *E. coli, C. difficile*	Endo-enterotoxemia	digestive	Caecum, descending & ascending colons	+ toxigenic (*tcdB*$^+$)
**37**	2019	August	Female	Adult	French Trotter	Normandy	14270		Unknown	Yes	Yes	Lung, lung abscess, left hock muscle, hock: *Klebsiella pneumoniae* , non-hemolytic *E. coli* , *Enterococcus* spp.; pool of digestive content: non-hemolytic *E. coli, Enterococcus faecalis*	Several concomitant infectious pathologies with pulmonary, musculoskeletal & digestive localization	locomotor & skeleton	Caecum, descending & ascending colons	+ toxigenic (*tcdB*$^+$)
**38**	2019	August	Male	Foal	French Trotter	Normandy	61230	~	Yes	Yes	No	Lung: *Rhodococcus equi* , non-hemolytic *E. coli* ; trachea, left hock, gastric lymph node abcesses: *Rhodococcus equi, Enterococcus* spp.	*Rhodococcus equi* infection	systemic	Caecum	-
**39**	2019	August	Female	Adult	Thoroughbred	Normandy	14340		No	Yes	No	No bacteriological analysis	Traumatic diaphragmatic rupture complicated by displacement of abdominal viscera	respiratory	Caecum	-
**40**	2019	August	Female	Yearlir	French Trotter	Normandy	76630		Unknown	No	No	No bacteriological analysis	Severe osteo-articular lesions of both stifles	locomotor & skeleton	Caecum	-
**41**	2019	September	Male	Foal	Thoroughbred	Normandy	14140	ù	No	Yes	No	No bacteriological analysis	volvulus of small intestine and caecum	digestive	Caecum	-

**42**	2019	September	Female	Adult	Pony	Normandy	14430	^	No	No	Yes	Caecum, ascending colon, small intestine: non-hemolytic *E. coli, Aerococcus viridans, Enterococcus faecalis, Candida guillermondii* in addition to *Clostridium perfringens* in caecum and *Clostridium perfringens, Paeniclostridium sordelli* in small intestine; Food: non-hemolytic *E. coli, Aerococcus viridans, Candida guillermondii*	Endo-enterotoxemia	digestive	Caecum, ascending colon	+ toxigenic (*tcdA*$^{+}$ *tcdB*$^{+}$)
**43**	2019	September	Female	Adult	Pony	Normandy	14430	^	Unknown	No	Yes	Caecum, ascending & descending colons, small intestine: non-hemolytic *E. coli, Aerococcus viridans, Enterococcus faecalis* in addition to *Clostridium perfringens* in caecum, ascending & descending colon	Endo-enterotoxemia	digestive	Caecum, descending & ascending colons	+ toxigenic (*tcdA*$^{+}$ *tcdB*$^{+}$)
**44**	2019	September	Female	Foal	French Trotter	Normandy	14340		No	No	No	No bacteriological analysis	Small intestinal perforation	digestive	Caecum	-
**45**	2019	September	Female	Young	Thoroughbrec	Normandy	14250		Yes	No	No	Brain, spinal cord, lung, liver: *Staphylococcus aureus, Enterococcus* spp.	Systemic infection, myeloencephalitis	systemic	Caecum	-
**46**	2019	December	Female	Foal	Thoroughbrec	Normandy	14140	!	No	No	No	Small intestine content: non-hemolytic *E. coli, Enterococcus faecalis , Streptococcus zooepidemicus*	Small intestinal perforation	digestive	Caecum	-
**47**	2019	December	Male	Young	French Trotte	Normandy	na		Unknown	No	No	No bacteriological analysis	Compressive cervical myelopathy	locomotor & skeleton	Caecum	-
**48**	2020	Fevruary	Female	Adult	Thoroughbrec	Normandy	14400	£	Yes	No	Yes	Wound, lung, liver, kidney: non-hemolytic *E. coli, Enterococcus* spp in addition to *Streptococcus zooepidemicus* in lung, liver, kidney; small intestine: non-hemolytic *E. coli, Enterococcus faecalis* , *C. difficile*	Endo-enterotoxemia	digestive	Caecum, Small intestine	+ non-toxigenic, not preserved
**49**	2020	Fevruary	Male	Young	French Trotte	Normandy	50870		Yes	No	No	No bacteriological analysis	Severe and advanced valvular endocarditis	cardiovascular	Caecum	-
**50**	2020	May	Female	Yearlin	French Trotte	Normandy	61500	$	No	No	No	Guttural pouchs, lymphatic node, right mandible, soft palate: *Streptococcus equi* ssp. *equi*, non-hemolytic *E. coli, Enterococcus* spp., *Staphylococcus xylosus*	Asphyxia by compression of the upper airways (strangles)	respiratory	Caecum	-
**51**	2020	June	Female	Adult	Thoroughbrec	Normandy	61310		Yes	No	No	abdominal abscess: *Enterococcus* spp. non-hemolytic *E. coli, Streptococcus equisimilis, Staphylococcus aureus* (SARM)	Rupture of the stomach following fibrous adhesions	digestive	Caecum	-
**52**	2020	June	Female	Foal	French Trotte	Normandy	na		Unknown	No	No	Lung abscess: *Rhodococcus equi, Streptococcus zooepidemicus, Serratia odorifera, Enterococcus* spp.; pubis: *Streptococcus zooepidemicus, Enterococcus* spp.	Severe and advanced osteomyelitis of the pelvis	locomotor & skeleton	Caecum	-
**53**	2020	June	Female	Foal	Thoroughbrec	Normandy	14130		No	Yes	Yes	Digestive content: non-hemolytic *E. coli, Enterococcus faecalis, Clostridium perfringens, Paeniclostridium sordellii* ; descending colon: Rotavirus	Gastric ulcer perforation	digestive	Caecum, descending & ascending colons	+ toxigenic (*tcdA*$^{+}$ *tcdB*$^{+}$ *cdt*$^{+}$)
**54**	2020	June	Male	Young	French Trotte	Normandy	14430		No	No	No	No bacteriological analysis	Internal bleeding (mesentery) following acute torsion of ascending colon	cardiovascular	Caecum, descending & ascending colons	+ toxigenic (*tcdA*$^{+}$ *tcdB*$^{+}$ unknown [PCR cdt not performed]), not preserved
**55**	2020	June	Female	Foal	French Trotte	Normandy	61310		Yes	No	No	Descending colon: Rotavirus; digestive content: non-hemolytic *E. coli, Enterococcus faecalis*	Gastric ulcers perforation following small intestinal volvulus	digestive	Caecum, Intes	-
**56**	2020	June	Female	Yearlin	French Trotte	Normandy	14540		No	Yes	No	No bacteriological analysis	Fracture of basal cranial bones	locomotor & skeleton	Caecum	-
**57**	2020	June	Female	Foal	French Trotte	Normandy	61500		No	No	No	Small intestine : non-hemolytic *E. coli, Enterococcus faecalis, Proteus mirabilis, Streptococcus zooepidemicus*	Myopathy	locomotor & skeleton	Caecum, ascending colon	+ toxigenic (*tcdA*$^{+}$ *tcdB*$^{+}$)
**58**	2020	July	Male	Foal	Thoroughbrec	Normandy	14140		Yes	No	No	Lung, lung abscess: *Rhodococcus equi, Enterococcus* spp.	*Rhodococcus equi* infection	respiratory	Caecum	-
**59**	2020	July	Female	Foal	Thoroughbrec	Normandy	14130		No	No	No	Descending colon: Rotavirus	Small intestinal volvulus	digestive	Caecum, Small intestine	-
**60**	2020	July	Female	Foal	French Trotte	Normandy	14140		Yes	Yes	Yes	Intestinal content: Rotavirus	Acute enteritis	digestive	Caecum, ascending colon & small intestine	+ non-toxigenic (initial detection: toxigenic *tcdB*$^{+}$)
**61**	2020	July	Female	Foal	Thoroughbrec	Normandy	14430	*	Yes	Yes	No	No bacteriological analysis	Small intestinal volvulus & infarction	digestive	Caecum, Small intestine	+ non-toxigenic (initial detection: toxigenic *tcdA*$^{+}$ *tcdB*$^{+}$)

**62**	2020	July	Male	Foal	Thoroughbred	Normandy	61310		Unknown	Yes	No	Lung: non-hemolytic *E. coli, Enterococcus* spp.	Interstitial bronchopneumonia	respiratory	Caecum	-
**63**	2020	July	Male	Foal	French Trotter	Normandy	14700		Yes	No	No	Lung abscess, lymphatic node: *Rhodococcus equi* , non-hemolytic *E. coli, Proteus vulgaris, Enterococcus* spp.; lung: *Rhodococcus equi* , non-hemolytic *E. coli*	*Rhodococcus equi* infection	systemic	Caecum, descending & ascending colons	-
**64**	2020	July	Female	Foal	Thoroughbred	Normandy	14430	*	Yes	No	No	Lung: *Rhodococcus equi* ; Lymphatic node: *Rhodococcus equi* , non-hemolytic *E. coli, Enterococcus* spp.; lung abscess: *Rhodococcus equi, Enterococcus s* pp. ; PG: *Rhodococcus equi, Pseudomonas aeruginosa, Enterococcus* spp., non-hemolytic *E. coli*	*Rhodococcus equi* infection	respiratory	Caecum	-
**65**	2020	July	Male	Yearlir	French Trotter	Normandy	14810	@	No	Yes	No	No bacteriological analysis	Compressive cervical myelopathy	locomotor & skeleton	Caecum	-
**66**	2020	August	Unknov	Foal	Unknown	Unknown	na		No	No	Unknown	Unknown	Unknown	unknown	Intestine	-
**67**	2020	September	Gelding	Adult	Saddlebred hc	Normandy	14520		No	No	No	No bacteriological analysis	Not pathognomonic	systemic	Caecum	-
**68**	2020	September	Gelding	Adult	Pony	Normandy	61000		No	No	Yes	No bacteriological analysis	Endo-enterotoxemia	digestive	Caecum	-
**69**	2020	September	Gelding	Young	Pony	Normandy	27310		No	No	No	Lung, Liver, kidney: *Pasteurella pneumotropica,* non-hemolytic *E. coli, Enterococcus* spp. in addition to *Streptococcus zooepidemicus* in lung	Systemic infection, interstitial pneumonia	systemic	Caecum	-
**70**	2020	September	Gelding	Adult	French Saddle	Normandy	14670		Yes	Unknown	No	Endocardium, lung, liver, kidney: non-hemolytic *E. coli, Enterococcus* spp.	Hemorrhagic syndrome	cardiovascular	Caecum	-
**71**	2020	September	Female	Adult	French Saddle	Normandy	14140		No	Yes	No	No bacteriological analysis	Small intestinal volvulus	digestive	Caecum	-
**72**	2020	October	Gelding	Foal	Irish Cob	Normandy	14240		No	No	No	Lung, lymphatic node: *Streptococcus zooepidemicus, Enterococcus* spp.	Compressive cervical myelopathy	locomotor & skeleton	Caecum	-
**73**	2020	October	Male	Young	French Trotter	Normandy	50300		Unknown	No	No	No bacteriological analysis	Atypical myopathy	locomotor & skeleton	Caecum	-
**74**	2020	November	Male	Yearlir	Thoroughbred	Normandy	14270		Yes	No	Yes	Lung, Liver, kidney: non-hemolytic *E. coli, Enterococcus* spp. ; digestive content: *Enterococcus faecalis* , non-hemolytic *E. coli* ; colons: *Clostridium perfringens*	Endo-enterotoxemia	digestive	Caecum, ascending colon	-
**75**	2020	November	Male	Foal	French Trotter	Normandy	61240		No	Yes	No	No bacteriological analysis	Degenerative myopathy	locomotor & skeleton	Caecum	-
**76**	2020	November	Female	Foal	Thoroughbred	Normandy	61470		Yes	No	Yes	Lung abscess: *Staphylococcus aureus, Pseudomonas* spp.	Endo-enterotoxemia	digestive	Caecum, ascending	-
**77**	2020	November	Female	Yearlir	Thoroughbred	Normandy	14430	*	No	Yes	No	No bacteriological analysis	Pelvic fracture	locomotor & skeleton	Caecum	-
**78**	2020	November	Female	Adult	Saddlebred hc	Normandy	27410		Yes	No	Yes	Absence of *Salmonella* spp.	Larval cyathostominosis	digestive	Caecum, ascending colon	+ toxigenic (*tcdA* $^{+}$ *tcdB* $^{+}$)
**79**	2020	November	Female	Adult	Thoroughbred	Normandy	61310		No	No	No	lymphatic node: *Enterococcus* spp., *Klebsiella pneumoniae* ssp. *ozaenae*	Rupture of the small intestine	digestive	Caecum	-
**80**	2020	December	Female	Adult	Donkey	Normandy	61340		No	No	Yes	Caecocolic content: non-hemolytic *E. coli, Salmonella enteritidis, Clostridium perfringens* ; ascending colon: non-hemolytic *E. coli, Enterococcus faecalis, Streptococcus zooepidemicus*	Endo-enterotoxemia	digestive	Caecum, descending & ascending colons	-
**81**	2020	December	Gelding	Adult	Irish Cob	Normandy	14100	§	No	No	Yes	Digestive content: *E.coli* ; small intestine abscess: non-hemolytic *E. coli, Klebsiella pneumoniae, Streptococcus zooepidemicus, Enterococcus faecalis* ; liver: *Enterococcus* spp., *Streptococcus zooepidemicus,*	Intestinal lymphoma	digestive	Caecum, descending & ascending colons	+ toxigenic (*tcdA* $^{+}$ *tcdB* $^{+}$)
**82**	2020	December	Female	Adult	Pony	Normandy	14430		No	No	No	Spleen: *Babesia caballi*	Rupture of the spleen	cardiovascular	Caecum	-
**83**	2020	December	Female	Foal	French Saddle	Normandy	14700		Yes	No	No	Fetlock: *Aerococcus viridans,* non-hemolytic *E. coli, Enterococcus* spp., *Saccharomyces cerevisiae*	Post-infectious necrotizing systemic vasculitis (hemorrhagic purpura)	cardiovascular	Caecum	-
**84**	2021	January	Female	Adult	Thoroughbred	Normandy	14950		No	No	No	No bacteriological analysis	Rupture of the vaginal artery	cardiovascular	Caecum	-
**85**	2021	January	Male	Foal	French Saddle	Normandy	50680		Yes	Yes	No	Paratesticular abscess: non-hemolytic *E. coli, Streptococcus zooepidemicus*	Duodenum ulcer perforation	digestive	Caecum	+ toxigenic (*tcdA* $^{+}$ *tcdB* $^{+}$)
**86**	2021	January	Female	Adult	French Trotter	Brittany	35370		No	No	No	No bacteriological analysis	Grass sickness	nervous	Caecum	-
**87**	2021	Fevruary	Female	Adult	Thoroughbred	Normandy	14140	ù	No	Yes	No	No bacteriological analysis	Eosinophilic enterocolitis	digestive	Caecum, ascending colon	-
**88**	2021	Fevruary	Female	Adult	Thoroughbred	Normandy	61240	}	No	No	No	No bacteriological analysis	Dystocic foaling	urogenital	Caecum	-
**89**	2021	Fevruary	Female	Adult	Thoroughbred	Normandy	14100		No	Yes	No	No bacteriological analysis	Rupture of the uterine artery	cardiovascular	Caecum	-

**90**	2021	March	Female	Yearlir	Thoroughbrec	Normandy	14140		No	Yes	No	No bacteriological analysis	Small intestinal perforation	digestive	Caecum	+ toxigenic (*tcdA* [+] *tcdB* [+])
**91**	2021	March	Female	Foal	Thoroughbrec	Normandy	61240	}	No	Yes	No	Lung, Liver, kidney, guttural pockets: *Enterococcus* spp.; small intestine content: non-hemolytic *E. coli, Enterococcus faecalis*	Systemic infection	systemic	Caecum	+ non-toxigenic
**92**	2021	April	Female	Foal	Thoroughbrec	Normandy	14100		No	No	No	Lung, Liver, kidney, right stifle: *Staphylococcus aureus, Streptococcus zooepidemicus* in addition to *Enterococcus* spp. in right stifle	Severe and extensive pleuropneumonia	respiratory	Caecum	+ toxigenic (*tcdB* [+])
**93**	2021	April	Female	Foal	Unknown	Normandy	76690		No	No	No	*Streptococcus equi* ssp. *equi*	Atypical form of strangles with sero-fibrinous pleurisy	respiratory	Caecum	-
**94**	2021	May	Female	Adult	French Trotte	Normandy	61470		Yes	No	No	*Clostridium perfringens, Paeniclostridium sordellii*	Myonecrosis	locomotor & skeleton	Caecum	-
**95**	2021	June	Female	Foal	Thoroughbrec	Normandy	14400	£	Yes	Unknowr	No	Non-hemolytic *E. coli*	Perforation of the descending colon	digestive	Caecum	-
**96**	2021	June	Female	Adult	Saddlebred hc	Normandy	61240		No	No	No	No bacteriological analysis	Rhinopneumonia (nervous form)	nervous	Caecum	-
**97**	2021	June	Female	Yearlir	Thoroughbrec	Normandy	14140	ù	Yes	No	No	Small inestine : non-hemolytic *E. coli, Enterococcus*	Ileocaecal intussusception	digestive	Caecum	-
**98**	2021	June	Female	Foal	Thoroughbrec	Normandy	14140		No	No	No	No bacteriological analysis	Small intestinal perforation	digestive	Caecum	-
**99**	2021	July	Male	Foal	French chaser	Normandy	61250		Yes	Yes	No	Lung abscess, caecum, ascending colon: *Rhodococcus equi,* non-hemolytic *E. coli* ; respiratory swab: *Rhodococcus equi, Citrobacter freundii*	*Rhodococcus equi* infection	systemic	Caecum	-
**100**	2021	July	Female	Foal	Thoroughbrec	Normandy	14810	@	No	Yes	Yes	*Rhodococcus equi* ; pool of digestive content: non-hemolytic *E. coli* ; small intestine: *Clostridium perfringens*	Acute typhlocolitis & *Rhodococcus equi* infection	digestive	Caecum, ascending colon	-
**Pilot study 2018**																
**0**	2018	September	Female	Foal	French Saddlebred Horse	Normandy	14400		Yes		Yes	Ascending colon: *Clostridium difficile,* non-hemolytic *Escherichia coli, Enterococcus faecalis* ; lung, guttural pouch: *Klebsiella pneumoniae*	Endo-enterotoxemia	digestive	ascending colon	+ toxigenic (*tcdB* [+])

All available data of each Equidae under study are shown.

Location: In 11 cases, several animals originated from the same premises (all 100 animals from 2019 to 2021 were from 78 different ones).

In the column 'Same premises', the following symbols: §, *, !, ù, }, µ, ^, $, ~, £ or @ are used to show all animals from each of the 11 premises having provided several animals.
